# The methylimidazolium ionic liquid M8OI is detectable in human sera and is subject to biliary excretion in perfused human liver

**DOI:** 10.1016/j.tox.2021.152854

**Published:** 2021-07

**Authors:** Alistair C. Leitch, Ibrahim Ibrahim, Tarek M. Abdelghany, Alex Charlton, Clair Roper, Dan Vidler, Jeremy M. Palmer, Colin Wilson, David E. Jones, Peter G. Blain, Matthew C. Wright

**Affiliations:** a Institute of Translation and Clinical Research, Newcastle University, Newcastle Upon Tyne, NE2 4AA, United Kingdom; b Department of Pharmacology and Toxicology, Faculty of Pharmacy, Cairo University, Kasr El-Aini St., Cairo, 11562, Egypt; c Freeman Hospital, Newcastle upon Tyne, Tyne and Wear, NE7 7DN, United Kingdom; d School of Natural and Environmental Sciences, Bedson Building, Newcastle University, NE1 8QB, United Kingdom

**Keywords:** ALP, alkaline phosphatase, ALT, alanine aminotransferase, AMA, anti-mitochondrial antibody, COOH7IM, 1-(7-carboxyheptyl)-3-methyl-1H-imidazol-3-ium, CYP, cytochrome P450, DBD, donation after brain death, DCD, donation after circulatory death, FMO, flavin-containing monooxygenases, HO8IM, 1-(8-hydroxyoctyl)-3-methyl-imidazolium, M8OI, 1-octyl 3-methylimidazolium chloride salt, M8OI^+^, 1-octyl 3-methylimidazolium cation, MI^+^, methylimidazolium cation, PBC, primary biliary cholangitis, PBS, phosphate buffered saline, PDC, pyruvate dehydrogenase complex, PFOA, perfluorooctanoic acid, PFOS, perfluorooctanesulfonic acid, Cytochromes P450, Alcohol dehydrogenase, Acetaldehyde dehydrogenase, Ionic liquids, Bile, C8mim

## Abstract

A methylimidizolium ionic liquid (M8OI) was recently found to be contaminating the environment and to be related to and/or potentially a component of an environmental trigger for the autoimmune liver disease primary biliary cholangitis (PBC). The aims of this study were to investigate human exposure to M8OI, hepatic metabolism and excretion. PBC patient and control sera were screened for the presence of M8OI. Human livers were perfused with 50μM M8OI in a closed circuit and its hepatic disposition examined. Metabolism was examined in cultured human hepatocytes and differentiated HepaRG cells by the addition of M8OI and metabolites in the range 10–100 μM. M8OI was detected in the sera from 5/20 PBC patients and 1/10 controls. In perfused livers, M8OI was cleared from the plasma with its appearance – primarily in the form of its hydroxylated (HO8IM) and carboxylated (COOH7IM) products – in the bile. Metabolism was reflected in cultured hepatocytes with HO8IM production inhibited by the cytochrome P450 inhibitor ketoconazole. Further oxidation of HO8IM to COOH7IM was sequentially inhibited by the alcohol and acetaldehyde dehydrogenase inhibitors 4-methyl pyrazole and disulfiram respectively. Hepatocytes from 1 donor failed to metabolise M8OI to COOH7IM over a 24 h period. These results demonstrate exposure to M8OI in the human population, monooxygenation by cytochromes P450 followed by alcohol and acetaldehyde dehydrogenase oxidation to a carboxylic acid that are excreted, in part, via the bile in human liver.

## Highlights

• M8OI was recently found to be contaminating the environment.
• M8OI was detected in the sera from 5/20 PBC patients and 1/10 controls.
• M8OI is taken up by human liver hepatocytes.
• M8OI is sequentially metabolised by CYPs followed by oxidation by dehydrogenases.
• The final carboxylic acid metabolite COOH7IM is, in part, excreted into human bile.

## 1 Introduction

PBC is a rare life-long chronic liver disease associated with high levels of anti-mitochondrial antibodies (AMA) in the sera of >95 % of PBC patients arising as a result of loss of immune tolerance to the lipoic acid-conjugated regions of the mitochondrial branched chain α keto acid dehydrogenase complexes (Hirschfield et al., 2018). The disease is more prevalent in women (by a factor of 10:1 (Griffiths et al., 2014)) and in particular in women over 50 (Hirschfield et al., 2018). However, male sex, younger age at onset (<45) and advanced disease at presentation are baseline predictors of poorer outcome (Hirschfield et al., 2018). The disease is characterised by chronic cholestatic inflammation, leading to hepatic portal tract fibrosis and a loss of intrahepatic bile ducts (Hirschfield and Gershwin, 2013). In a proportion of patients, inadequate responses to therapies (e.g. ursodeoxycholic acid) result in severe disease and a requirement for liver transplantation (Hirschfield et al., 2018).

The causes of PBC are unknown but a key factor is likely to be a genetic pre-disposition since incidence is higher in families (concordance rate of 63 % (5/8) and in monozygotic twins (Selmi et al., 2004). Several independent genome-wide association studies indicate that a variety of polymorphisms associated with inflammatory and immunological functions are weakly associated with PBC incidence but none of these singly or in combination are predictive in the way that a genetic mutation may be predictive of a genetically-inherited disease (Hirschfield et al., 2018). The absence of a predictive genetic signature for PBC suggests that incidence is significantly dependent on exposure to an environmental factor(s), but only in a relatively small proportion of those exposed to the factor(s). Such an environmental factor may be associated with deliberate exposure to widely used agents (e.g. component of cosmetics and hair dyes (Prince et al., 2010)) or to unintentional exposure to agents that are likely to be widespread (e.g. infectious agents (Haydon and Neuberger, 2000; Mason, 2018)) or subject to a degree of geographical variation (e.g. xenobiotics in the environment). The existence of xenobiotic triggers is supported by a 10-fold increase in PBC incidence associated with a drinking water source (Triger, 1980); spatial clustering in a heavily mined region (Prince et al., 2001; Dyson et al., 2020) and increased prevalence near Superfund toxic waste sites (Ala et al., 2006).

Recent research identified a xenobiotic present in soils around a landfill in the north east of England, initially on the basis of its ability to induce the apoptosis of a liver progenitor cell (Probert et al., 2018). The xenobiotic was identified as the ionic liquid 1 octyl 3 methylimidazolium (also known as 1-methyl-3-octyl imidazolium; C8mim or M8OI). M8OI in the environment could have been derived from 1 of up to 5 commercially-available EU-registered octyl methylimidazolium ionic liquids which themselves belong to a group of 64 structurally-related EU-registered methylimidazolium ionic liquids [ECHA website: https://echa.europa.eu/]. They are man-made environmentally-persistent chemicals currently used in the transfer of chemicals (e.g. storing and transporting toxic gases which are widely used in the electronics industry to dope silicon (Greer et al., 2020)); in formulation of mixtures and formulation in materials; in thermoplastic manufacture; in processing aids at industrial sites and in adhesives and sealants, coating products and inks/toners. In addition, they are being proposed as “green” replacements for widely-used volatile solvents (Amde et al., 2015; Xiao et al., 2018; Leitch et al., 2020a).

Only limited data are publicly available regarding M8OI and potential adverse effects in mammalian systems. In short term studies in mice using an i.p. route of exposure, hepatic changes were limited to a significant dose-dependent loss of hepatic glycogen and a mild but significant increase in portal tract inflammatory recruitment and/or fibroblastic proliferation accompanied by a focal fibrotic change (Leitch et al., 2020b). However, the kidney was the key target organ in our hands, with focal and mild to multifocal and moderate degeneration with a general trend for an increase in severity with increased dose. These changes were accompanied by a dose-dependent increased expression of Kim1 in kidney tissue, marked elevations in urinary Kim1 protein and a dose-dependent increase in serum creatinine (Leitch et al., 2020b). Interestingly, renal injury is also seen in PBC patients (Kamouchi et al., 1991). In vitro studies with cells indicate that M8OI and similar methylimidzolium ionic liquids inhibit oxidative phosphorylation through 2 separately-distinct interactions, leading to an induction of apoptosis (Abdelghany et al., 2020). With M8OI, oxygen consumption by cells is rapidly inhibited, preceding caspase 3/7 induction and DNA fragmentation. The block in oxygen consumption is ostensibly due to an acceptance by M8OI of electrons from the mitochondrial electron transport chain, redox cycling and reactive oxygen species production since there is no direct inhibition of mitochondrial complexes I-IV or complex V (F_0_F_1_-ATPase) by M8OI (Abdelghany et al., 2020). The block in mitochondrial function likely accounts for renal sensitivity to systemic M8OI, given the kidney’s high oxygen consumption.

Methylimidazolium ionic liquids are not currently included on the lists of a variety of organisations that routinely perform environmental or biomonitoring assessments [e.g. CDC’s National Biomonitoring Program (NBP) https://www.cdc.gov/biomonitoring/environmental_chemicals.html; see also https://www.hbm4eu.eu/]. It is noted however, that an exclusively-used ionic liquid (including methylimidazolium ionic liquids) anion, tris(pentafluoroethyl)trifluorophosphate, has recently been detected in 3 rivers in Germany (Neuwald et al., 2020).

There are a number of attractive aspects with methylimidazolium ionic liquids and their potential to act as triggers for PBC. Considering M8OI, the chemical is toxic to cells through interactions with the mitochondrial electron transport chain (Abdelghany et al., 2020), leading to the induction of apoptosis. M8OI is also an activator of the human estrogen receptor alpha (Leitch et al., 2018), which is associated with both cholestasis (Stieger et al., 2000; Yamamoto et al., 2006) and the exposure of non-glutathionylated PDC-E2 on the surface of cells (Hu et al., 2012). The latter of these is thought to be essential in the development of PBC and AMA (Lleo et al., 2009; Kawata et al., 2012; Chang et al., 2018). Finally, human hepatocytes metabolise M8OI to a carboxylic acid metabolite that is capable of enzymatic incorporation into PDC-E2 in place of lipoic acid (Probert et al., 2018).

To conclude on the theoretical potential that M8OI could trigger PBC, we hypothesised that exposure to M8OI is occurring in the general population; that M8OI is taken up by the liver and both a proportion of M8OI and its carboxylic acid metabolite 1-(7-carboxyheptyl)-3-methyl-1H-imidazol-3-ium (COOH7IM) are excreted into the bile. To test this hypothesis, the presence of M8OI in sera from PBC patients and controls has been examined. The disposition of M8OI and its metabolites has also been examined in perfused human liver in addition to an investigation into the enzymes mediating its hepatic metabolism.

We demonstrate for the first time, evidence for exposure to M8OI in the general population; demonstrate that a proportion of M8OI and COOH7IM in liver perfusates is excreted in bile and that production of COOH7IM is dependent on cytochromes P450 monooxygenation followed by alcohol and acetaldehyde dehydrogenase oxidation.

## 2 Materials and methods

### 2.1 Chemicals

3-methyl-1-octyl-1H-imidazol-3-ium (M8OI) was purchased from Sigma (Poole, UK). 1-(7-carboxyheptyl)-3-methyl-1H-imidazol-3-ium (COOH7IM) was custom synthesized with purity and chemical structures determined by HPLC, mass spectrometry and NMR techniques (Probert et al., 2018). 1-(8-hydroxyoctyl)-3-methyl-imidazolium (HO8IM) was also custom synthesised, with chemical structure confirmed (see Supplementary Fig. 1).

### 2.2 PBC patient and control serum samples

Twenty serum samples from PBC patients and 10 control serum samples were obtained via the UK-PBC consortium (http://www.uk-pbc.com/). Access to samples was subject to ethical review as outlined (http://www.uk-pbc.com/resources/data-access/).

### 2.3 Serum AMA determination

Quantitation of anti-PDC specific autoantibodies was determined using a well-established in-house ELISA (Heseltine et al., 1990). Highly purified bovine PDC was used as coating antigen on Immulon 4HBX 96 well microtitre plates (5 μg/mL). Detection of bound antibodies was detected with goat anti-human IgG peroxidase conjugated antibody (Sigma). Bound peroxidase activity was visualised using o-phenylenediamine and measured at 492 nm. Under standard ELISA conditions, the mean absorption value (492 nm) from the sera of 80 healthy volunteers at a dilution of (1:1000) was 0.058 ± 0.025 (mean ± SD). Values greater than 2 SD above the mean (0.108) were defined as positive. The "absolute antibody titre" was defined as the maximal dilution of a serum sample at which the ELISA still proved positive.

### 2.4 Serum M8OI determination

Serum samples were defrosted and diluted 1:1 (v/v) in 1 % formic acid, followed by centrifugation at 13,000 rpm for 5 min. Supernatants were retained and the presence of M8OI and metabolites determined using an LC-QTOF-MS system. This comprised a Shimadzu XR UHPLC system coupled to a Sciex TripleTOF 5600+ mass spectrometer equipped with an ESI (Electrospray Ionisation) Turbo Spray ion source. The Shimadzu UHPLC system included two pump modules (model LC-20ADXR), a degassing unit (DGU-20A3R), an autosampler (model SIL-20ACXR), a column oven (model CTO-20AC) and a communications bus module (model CBM-20A). The analytical LC column used was a Raptor ™ Biphenyl, 2.7 μm particle size, 100 mm length x2.1 mm internal diameter (Restek, USA) equipped with guard column containing the same chromatographic phase, 5 mm length x2.1 mm internal diameter. The column oven held analytical LC column and guard column at 50 °C for the analysis, and the autosampler tray temperature was set to 15 °C. A mobile phase flow rate of 0.4 mL/min and a sample injection volume of 2 μL were employed. This binary flow UHPLC gradient was made from mixing 0.1 % formic acid in water (mobile phase “A”) and 0.1 % formic acid in methanol (mobile phase “B”). Initial mobile phase composition was 95 % mobile phase “A” and 5 % mobile phase “B”, followed by a linear gradient to 95 % “B” over 8 min, and then held at 95 % “B” for 2 min. The mobile phase was then returned to the initial mobile phase composition for 3 min prior to the next sample injection.

Due to the nature of M8OI, only positive mode ESI mass spectrometric data were acquired. As we were only interested in the presence or absence of M8OI in the sample set, product ion spectra from a precursor ion at *m*/*z* 195.2 were acquired. MS data were acquired using a duty cycle that comprised a “TOF MS” experiment followed by a “Product Ion” experiment. The collision energy (CE) was set to a value of 10 V for the low energy TOF MS experiment and the mass range used was *m*/*z* 120 to *m*/*z* 300. The CE was set to 30 V for the Product Ion experiment and the *m*/*z* range acquired for product ions derived from *m*/*z* 195.2 was from *m*/*z* 30 to *m*/*z* 230 in High Resolution mode. ESI source conditions were the same throughout: Ion Source Gas 1 (GS1) – 45 psi, Ion Source Gas 2 (GS2) – 35 psi, Curtain Gas (CUR) – 45 psi, Temperature (TEM) – 450 °C, Ion Spray Voltage Floating (ISVF) – 5500 V and Declustering Potential (DP) – 80 V. Sciex Analyst instrument control software (Analyst TF 1.7.1) was used to control both the Shimadzu UHPLC and TripleTOF mass spectrometer, and to acquire MS data. Prior to running a sample batch the QTOF-MS was tuned and calibrated following the manufacturer’s instructions. Within batch mass auto-calibration was achieved by injecting a standard solution every 5 sample injections to perform MS and MS/MS mass calibration on-the-fly. Samples were bracketed with blank injections to establish whether any measurable carry-over was observed over the course of a sample batch. Mass spectrometric data were visualised in PeakView (Version 2.2, Sciex).

### 2.5 Liver perfusion

Consent for recruitment of livers into a research study was obtained from the donor’s next of kin by specialist nurses in organ donation with allocation overseen by the NHS Blood and Transplant’s Research Innovation and Novel Technologies Advisory Group. Organs undergoing normothermic machine perfusion were part of an approved project (IRAS number 179433, NHS Blood and Transplant liver study 52, NuTH R&D study 7483) to examine graft viability and therapeutics having the potential to reduce graft viability. Potentially transplantable livers were flushed and perfused in situ with ice-cooled University of Wisconsin preservation solution (ViaSpan), then surgically removed with cannulation of the hepatic artery, portal vein, inferior vena cavae and common bile duct. When deemed non-transplantable, livers were transported to the Freeman Hospital (Newcastle Upon Tyne, UK) on ice and the preservation solution was flushed out of the organ with 0.9 % (w/v) NaCl followed by connection to a custom-built circuit consisting of CARMEDA® BioActive Surface heparin-tubing, a Medtronic Bio-Console 560 Speed Pump Controller and Hirtz Hico-Variotherm 555 heater-cooler (see Fig. 2a). The graft was then perfused at 37 °C with oxygenated human erythrocyte-based perfusate (3 units of type-specific human packed red blood cells, 10,000 units of unfractionated heparin, 10 mL of 10 % calcium gluconate, 500 mL of Isoplex 4 % w/v (succinylated gelatin) solution, 8.4 % w/v sodium bicarbonate – giving an approximate total initial perfusate volume of 1.25–1.5 L). The circuit setup allows dual perfusion of the graft via the hepatic artery and portal vein in a closed circuit setting (hepatic artery, portal vein, bottom opening inferior vein cava and common bile duct are cannulated). Perfusion pressures in the hepatic artery and portal vein were maintained at 70 mm and 5 mm respectively using a pinch valves applied to the inflow tubing of the portal vein. Periodic injections of heparin, Flolan (epoprostenol sodium), Actrapid (recombinant human insulin), Synthamin 9 (5.5 % amino acids infusion), and Cernevit (multivitamins fir infusion) were made when required. Blood gas analysis was employed to monitor perfusate pO_2_ and pH with additions of sodium bicarbonate and oxygen to assist in maintaining pH > 7.2 and pO_2_ > 15kPA respectively. After graft validation, M8OI (final perfusate concentration approx. 50 μM, chosen for ease of detection by all available methodologies available, was added to the blood perfusate via the inflow limb by removing 50 mL s of perfusate and mixing with M8OI prior to return to the perfusion circuit. The liver was perfused for a further 3 h during which time, perfusate was sampled from both the arterial and venous limbs of the circuit in addition to regular sampling from the bile.

### 2.6 Clinical chemistry and other perfusate parameters

Perfusate pH, oxygen saturation (pO2), glucose and lactate concentrations and other parameters (Na^+^, K^+^, Ca^2+^, Cl–, Haematocrit, total haemaglobin (tHb), oxygenated haemaglobin (O_2_Hb), carboxyhaemaglobin (COHb), methaemaglobin (MetHb), deoxygenated haemaglobin (HHb) and oxygen saturation) were determined using a GEM Premier 5000 blood gas testing system (Werfen,Warrington, UK). Oxygen consumption (mL/min/kg liver) was calculated based on the difference between the venous and arterial oxygen contents of the circulating perfusate, by adding the free dissolved oxygen fraction (pO_2_*K) to the haemaglobin (Hb)-bound oxygen fractions (sO_2_ *Hb*c) using the following formula: O_2_ content = (pO_2_*K) + (sO_2_ *Hb*c), where pO_2_ is partial oxygen pressure in kPa; K equals 0.027 for O_2_ in water at 37 °C; sO_2_ is the saturation expressed as a fraction; Hb is the concentration in mmol/L and c equals 91.12 mL O_2_/mmol for the oxygen binding capacity of Hb. Perfusate samples were aspirated from both the arterial and venous limbs of the circuit, centrifuged to pellet erythrocytes and the supernatant was aliquoted and stored at −20 °C prior to analysis for alanine aminotransferase (ALT) and alkaline phosphatase (ALP), determined essentially as previously described (Marek et al., 2005). Temperature and perfusion pump speeds were continuously monitored.

### 2.7 Primary hepatocyte isolation and culture

Human liver was ethically obtained via the Newcastle Transplant Tissue Biobank [Newcastle and North Tyneside Research Ethics Committee 1 (5979, REC:17/NE/0022)], with specific approval from a local Research Ethics Committee for these specific studies. Discarded human liver grafts offered for research were reconditioned by normothermic machine perfusion with whole human blood over a period of around 24 h. After reconditioning by normothermic machine perfusion with whole human blood as outlined above, some livers were subsequently used directly for cell isolations. In these cases, the liver was split to allow for fully cannulated perfusion (at 150 mL/min @ 37 °C) of the chosen left lateral segment of the liver. Human hepatocytes were then isolated by a 2 step collagenase perfusion (with collagenase step re-circulated until digestion, typically 20–30 min s) and cultured essentially as previously described (Harvey et al., 2000) in Williams medium E supplemented with 10 % (v/v) FCS, 80 u/mL penicillin, 80 μg/mL streptomycin, 10 nM dexamethasone and 1ug/mL insulin. After an overnight culture period, the medium was aspirated, the cells were washed 3 times with sterile PBS and subsequently cultured in fresh medium without serum, with a daily change thereafter.

### 2.8 HepaRG cell culture

HepaRG cells were obtained from INSERM Transfert SA (Biopark Paris, France via Biopredic) and routinely expanded in Williams' E medium supplemented with 10 % (v/v) FCS, 100 units/mL penicillin & 100 μg/mL streptomycin, 5 μg/mL insulin and 500 nM hydrocortisone hemisuccinate sodium salt. The cells were differentiated into a mixed culture of hepatocyte-like and cholangiocyte-like cells (Aninat et al., 2006) through increasing the concentration of hydrocortisone hemisuccinate sodium salt to 50 μM.

### 2.9 M8OI metabolism (LC-HR-MS/MS)

The metabolism of M8OI was determined in perfusate serum and bile by LC-HR-MS/MS techniques as previously outlined (Probert et al., 2018) using a TripleTOF 5600 high-resolution quadrupole time-of-flight (TOF) mass spectrometer (Sciex) equipped with a DuoSpray ion source operated in positive electrospray mode, coupled to an Eksigent Nano LC 420 system. Metabolism in cultured hepatocytes and HepaRG cells was performed using STIM buffer (0.10 M NaCl, 5.4 mM KCl, 0.34 mM Na2HPO4 12H2O, 0.44 mM KH2PO4, 20 mM glucose, 1 mM CaCl2, 40 mM NaHCO3, 4 mM glutamine, 100μM l-alanine, 100μM l-asparagine, 100μM l-aspartic acid, 100μM l-glutamic Acid, 100μM glycine, 100μM l-proline and 100μM l-serine, pH 7.4 when gassed with 5 % CO_2_ in air).

### 2.10 M8OI metabolism (HPLC, rapid determination)

When sufficient cells were available from donors, a rapid assessment of M8OI metabolism was performed using high concentrations of M8OI and an HPLC with UV detection. The higher concentrations used enabled the metabolic capacity to be determined using this less sensitive methodology, which was routinely available for rapid determinations. This rapid turnaround also enabled decisions to be made regarding experiments with cells, which remain differentiated for a short time only (Wallace et al., 2010). Freshly isolated hepatocytes were cultured overnight to allow for attachment, washed and cultured in STIM buffer supplemented with M8OI. Samples of this incubation were taken at various times and 1 vol of 1 % HPLC-grade phosphoric acid was added to 10 volumes of sample. The sample was vortexed and centrifuged for 1 min at 16,000 *g*. The supernatant was transferred to HPLC vials (Thermo Scientific) and subjected to HPLC using an LC-20AD system with Nucleosil C18 column (25 cm x3.2 mm). Samples were eluted with a mobile phase gradient system of (A) 0.1 % phosphoric acid (Fisher) and (B) acetonitrile (Fisher) at a flow rate of 0.7 mL/min [0 min: A = 90 % / B = 10 %; 15 min: A = 10 % / B = 90 %; 30 min: A = 10 % / B = 90 % and 35 min: A = 90 % / B = 10 %]. M8OI and COOH7IM were detected at 211 nm and concentrations were determined using authentic standard.

### 2.11 Statistics

The student’s two-tailed T-test was used to determine significant difference between groups. Significance was achieved where p < 0.05. For comparison of multiple groups, ANOVA was carried out and where significant, differences between groups were determined using Bonferroni-Holm method. Where p < 0.05, a significant difference was assumed.

## 3 Results

### 3.1 M8OI is present in PBC patient sera

Although M8OI has been detected in soils around a landfill site in the north east of England, it is not known whether this contamination is unique to this area, since methylimidazolium ionic liquids are not currently included on the lists of a variety of organisations that routinely perform environmental or biomonitoring assessments. A limited pilot biomonitoring study was therefore undertaken to establish whether there was any evidence for exposure in the human population. Given our proposal that, in theory, M8OI could be a hazard trigger for PBC, exposure in 20 PBC patients was examined and compared to 10 controls.

**Fig. 1:**
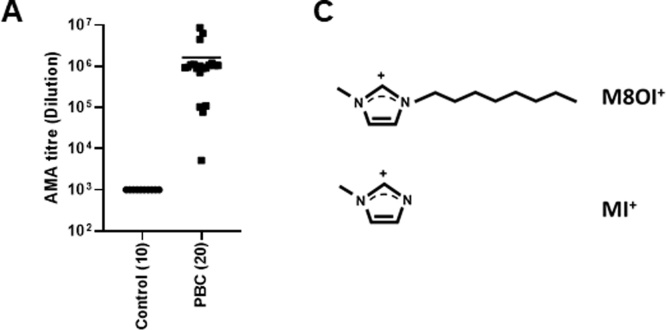

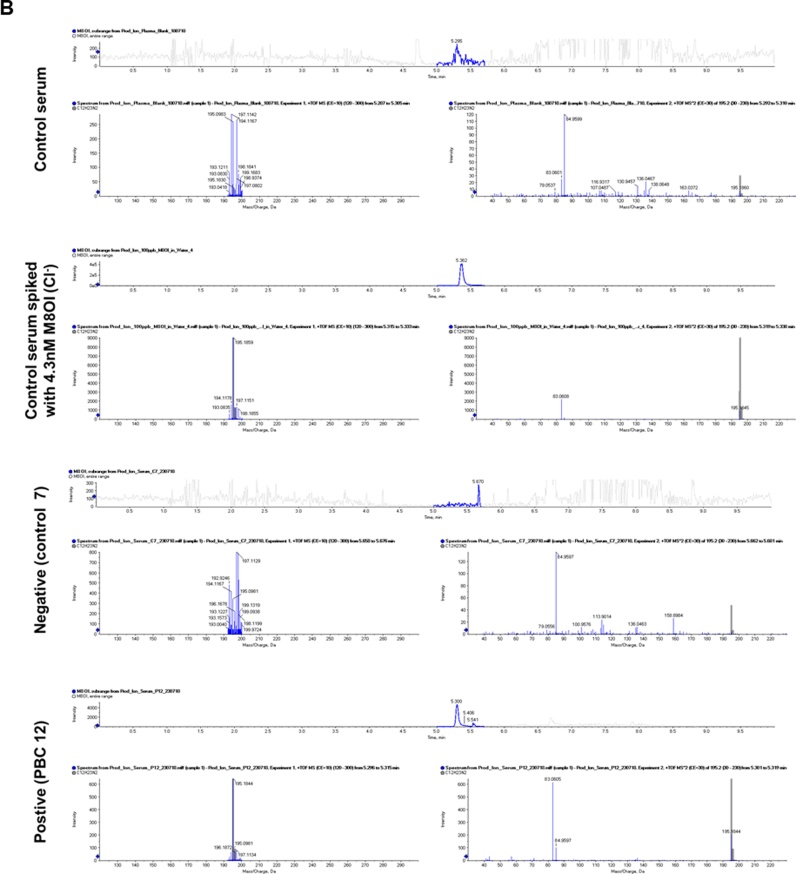
**The M8OI cation is detectable in sera from PBC patients**. **A**, individual serum AMA titres for control and PBC serum samples. **B**, LC and MSMS data for the indicated serum samples. Control serum spiked with 4.3 nM M8OI^+^ (1 ng/mL M8OI Cl^−^ salt); negative, a serum sample considered to be lacking detectable levels of M8OI^+^; positive, a serum sample considered to be containing M8OI^+^. **C**, Upper structure, the M8OI cation (M8OI^+^). According to Chemspider (http://www.chemspider.com/), the monoisotopic mass of the M8OI chloride salt (C_12_H_23_ClN_2_; ChemSpider ID2015979) is 230.154984 Da. Using a monoisotopic mass for the chlorine atom of 34.968853 Da (Cl^o^ ; ChemSpider ID4514529), this indicates that the monoisotopic mass for the M8OI cation (M8OI^+^) is 195.1861. Lower structure, methylimidazolium cation (MI^+^). According to Chemspider, the monoisotopic mass of MI^+^ (C_4_H_7_N_2_; ChemSpider ID392224) is 83.060371 Da.

Fig. 1a confirms that all PBC patients had variably elevated levels of serum AMA (titre 1.6 +/- 2.2 × 10^6^) compared to control (titre < 1 × 10^3^, defined as negative as ELISA reading was within 2 SD of reading from 80 healthy volunteers). Screening (and excluding all other fragments) for the presence of the intact M8OI^+^ cation identifies a single peak with a retention time of 5.3–5.4 minutes in a control serum spiked with M8OI. MS/MS of this peak confirms the mass of the intact cation (195.1845 Da) and a fragment with a mass of 83.0608 Da due to fragmentation to produce a methylimidazolium (MI^+^) fragment (Fig. 1b, upper panel) – see also Fig. 1c. In many of the serum samples, this analysis produced low levels of a fragment with a mass equivalent to the intact M8OI^+^ cation accompanied by a fragment in the region of 84.96 Da (Fig. 1b, middle panel). This pattern of fragmentation is likely associated with a normal constituent of sera and not M8OI. However, in 1 control and several PBC serum samples, the fragment pattern was identical to M8OI-spiked serum, containing fragments with a mass associated with both the intact M8OI^+^ and MI^+^ cations. Applying a cut-off that the abundance of the 83 peak must be at least 50 % higher than the abundance of the 84.96 peak for the serum sample to be considered to contain detectable levels of M8OI, this indicates that 1 out 10 control sera and 5 out of 20 PBC patient sera contained M8OI (Table 1).

**Fig. 2:**
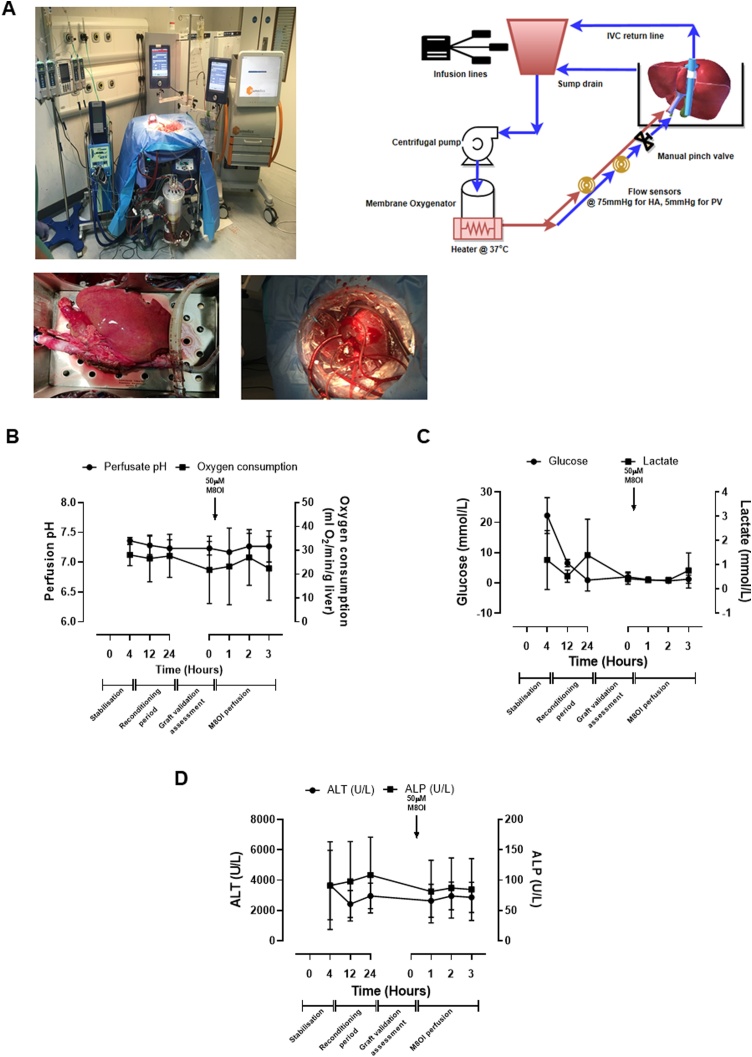
**Liver re-conditioning and effects of M8OI inclusion in the perfusate. A**, left panel, whole liver organ perfusion; lower left panel, close view of vessel cannulation in whole liver perfusion; lower right panel, cannulation for end of lobe collagenase perfusion. Right panel, schematic of perfusion system. Time course for changes in perfusate pH and pO2; (**B**) glucose and lactate (**C**) and ALT and ALP (**D**).

**Table 1:** Detection of M8OI + and MI + fragments in sera from control and PBC patients by LC MS/MS.

Sample ID	LC	MS	MS/MS	MS/MS
	AUC for RT 5.3 peak	M8OI^+^ fragment (195.1861)	MI^+^ fragment (83.0603)	Abundance of fragment 83 (MI^+^) > 50% abundance of fragment 84.96?
C1	1334	+	+	+
C2	223	+	–	–
C3	223	+	–	–
C4	223	–	–	–
C5	53	–	–	–
C6	53	+	+	–
C7	280	–	–	–
C8	104	+	–	–
C9	606	+	+	–
C10	201	+	–	–
PBC1	404	+	+	–
PBC2	447	+	+	–
PBC3	544	+	–	–
PBC4	284	–	–	–
PBC5	403	+	+	–
PBC6	1283	+	+	+
PBC7	380	+	+	–
PBC8	5503	+	+	+
PBC9	133	+	–	–
PBC10	546	+	+	+
PBC11	679	+	+	–
PBC12	4684	+	+	+
PBC13	326	+	+	–
PBC14	255	+	–	–
PBC15	679	+	+	–
PBC16	160	+	–	–
PBC17	406	+	–	–
PBC18	176	+	+	–
PBC19	639	+	+	+
PBC20	376	+	–	–
M8OI Cl^−^ authentic standard (1 ng/mL)	4440	+	+	+

+, present; -, absent.

These data suggest that there are detectable levels of M8OI in some individuals and therefore that there has been exposure to M8OI in the human population. However, it is notable that no evidence for the presence of the known metabolites of M8OI – HO8IM and COOH7IM – was observed in any sample. Although limited in the number of individuals examined, the identification of M8OI in sera from 5/20 PBC patients and 1/10 controls suggest that exposure to M8OI may be higher in PBC patients compared to controls.

### 3.2 M8OI is rapidly cleared from the perfusate, biotransformed and excreted into the bile during human liver perfusion

To determine whether M8OI is metabolised and excreted into the bile in man, following organ re-conditioning, intact whole livers (for donor details see Table 2), were exposed for 3 h with perfusate containing M8OI.

**Table 2:** Details of liver donors used in M8OI whole liver perfusions.

ID	Sex	Age	BMI	Drug history	Cause of death/donor type
Donor 1 (NMP05)	Male	52	26.8	Methylprednisolone, noradrenaline, vaspressin, labetolol.	Intracranial haemorrhage/DBD
Donor 2 (NMP06)	Male	71	28.6	Olanzapine, pregabalin, sertaline, mirtazapine, metronidazole, paracetamol, labetalol, tazocin, ranitidine, tinzaparin, mannitol 20 %.	Cardiac arrest/DCD
Donor 3 (NMP10)	Male	73	31.9	Alfuzosin, alverine, aspirin, atenolol, buprenorphine, c-careldopa, madopar, pramipexole, sinemet, stalevo, simvastatin, quinine sulfate, noradrenaline, insulin, metaraminol, propofol, keppra, sodium valproate, phosphate polyfusor, fentanyl, tazocin, co-amoxiclav, phenytoin.	Cardiac arrest/DCD

DCD, donation after circulatory death; DBD, donation after brain death.

**Fig. 3:**
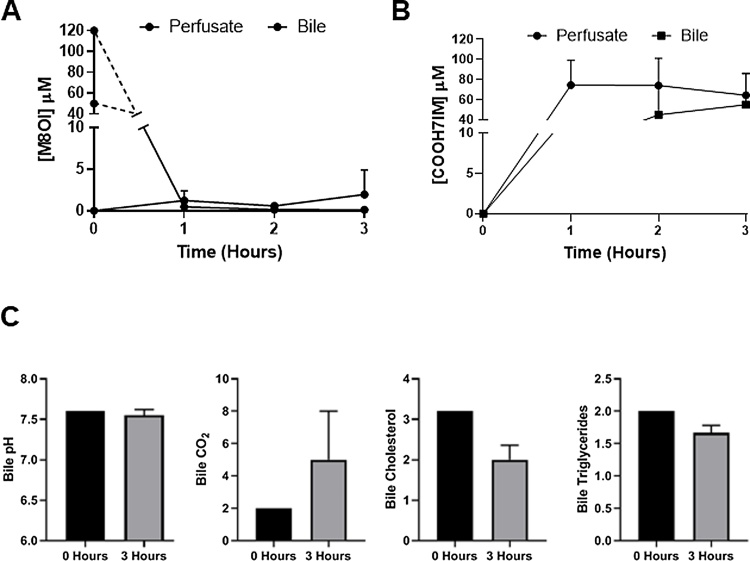
**M8OI is cleared from the perfusate, biotransformed with metabolite appearance in the perfusate and bile and changes in bile constituents in whole human liver perfusion. A**, Concentrations of M8OI in perfusate and bile after addition approx. 50μM M8OI (based on estimates of perfusion volume). **B**, Concentrations of COOH7IM in perfusate and bile. Data are the mean and SD of 3 separate perfusions. **D**, Biliary levels for the indicated endpoint at time zero and after 3 h perfusion with M8OI. Data are the mean and SD of 3 separate perfusions. Note, it is not possible to run a comparative control from the same donor liver. However, these markers were stable prior to addition of M8OI to the perfusate.

Fig. 2a-d demonstrate that a variety of perfusate endpoints (pH, pO_2_, glucose, lactate, ALT and ALP) were stable or were stabilised during re-conditioning. Furthermore, addition of M8OI did not result in any marked changes in these endpoints during the subsequent 3 h exposure, suggesting M8OI did not stress the liver or cause toxicity during this period. Fig. 3a demonstrates that M8OI was almost completely removed from the circulating perfusate within 1 h (the earliest time point repeatedly examined; a single 20 min perfusion time point from a donor showed a similar degree of uptake, data not included) and that very low levels of intact M8OI appeared in the bile. Low levels of the hydroxylated metabolite HO8IM also appeared in both the perfusate and bile (data not shown) with the majority appearing as the carboxylic acid metabolite COOH7IM (Fig. 3b). Despite an absence of any change in perfusate endpoints (Fig. 2), Fig. 3c suggest that perfusing the liver with M8OI modulated the bile constituents on the basis of CO_2_, cholesterol and triglyceride levels.

These data demonstrate that M8OI is removed from the hepatic perfusate and metabolised to a carboxylic acid metabolite that appears in both the perfusate and bile in the intact pefused human liver.

### 3.3 M8OI is primarily hydroxylated by a cytochrome P450(s) that is sensitive to ketoconazole inhibition in primary human hepatocytes

To determine the hepatic enzymes likely mediating the conversion of M8OI to HO8IM, M8OI disappearance and HO8IM appearance were examined in cultured human hepatocytes (see Table 3 for donor details). The metabolism of M8OI to HO8IM is a reaction typically catalysed by monooxygenases, therefore, the effect of inhibitors of the main hepatic monooxygenases – cytochromes P450 (CYP) and flavin-containing monooxygenases (FMO) were examined.

**Table 3:** Detail of liver donors used to prepare hepatocytes.

ID	Sex	Age	BMI	Drug history	Cause of death/donor type
Donor 1 (NHL1)	Male	23	30.8	Propofol, Fentanyl, Midazolam, Sertraline, Fluoxetine, Citalopram, Lansoprazole, Gentamicin, Metronidazole, Teicoplanin, Tetanus immunoglobulin, Naproxen, Co-codamol.	Road traffic accident
Donor 2 (NHL2)	Female	64	22.9	Dexamethasone, Meropenem, Levetiracetam.	Brain tumour
Donor 3 (NHL3)	Male	42		Propofol, Fentanyl, Midazolam, Amiodarone, Adalat, Loperamide.	
Donor 4 (NHL4)	Male	60	27.0	Propofol, Alfentanil, Insulin, Noradrenaline, Potassium chloride, Levetiracetam, Ticagrelor, Adrenaline, Amiodarone, Beclometasone dipropionate anhydrous and Formoterol fumarate dehydrate, Omeprazole, Aspirin.	Hypoxic brain damage
Donor 5 (NHL5)	Male	56	29.8	Noradrenaline, metaraminol, co-amoxiclav, tazocin, dalteparin, propafol, alfentanil.	Intracranial haemorrhage

**Fig. 4:**
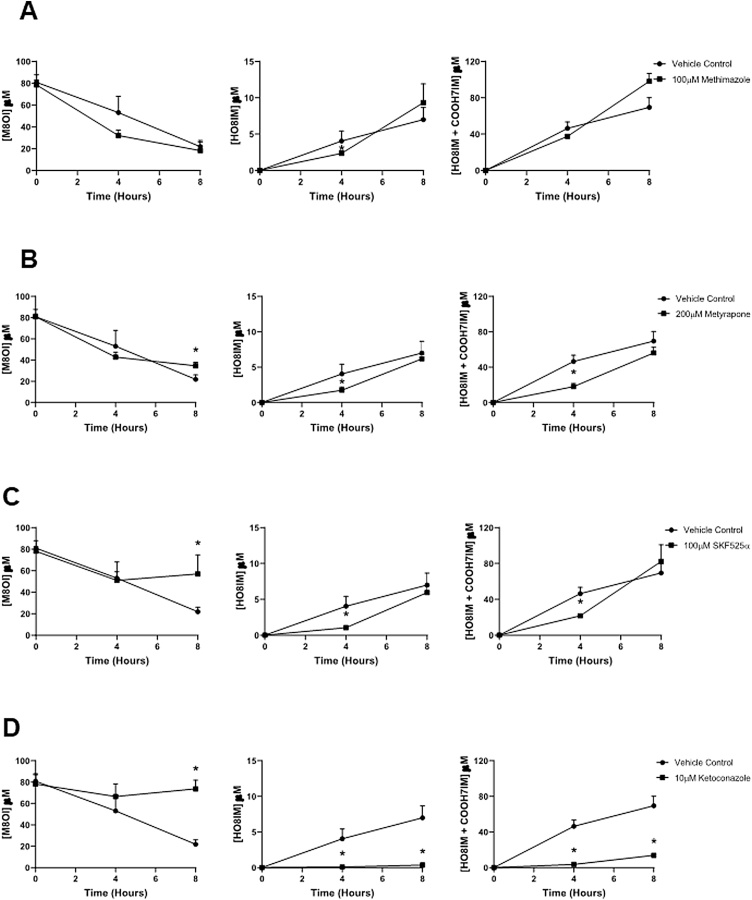
**M8OI is primarily hydroxylated by a cytochrome(s) P450 and not by a flavin-containing monooxygenase in human hepatocytes. A**, Medium concentrations of the indicated parent M8OI or metabolite(s) with time with or without the addition of 100 μM methimazole. **B**, Medium concentrations of the indicated parent M8OI or metabolite(s) with time with or without the addition of 200 μM metyrapone. **C**, Medium concentrations of the indicated parent M8OI or metabolite(s) with time with or without the addition of 100 μM SKF525a. **D**, Medium concentrations of the indicated parent M8OI or metabolite(s) with time with or without the addition of 10 μM ketoconazole. Data are the mean and SD of 3 separate cultures from the same donor, qualitatively representative of cells from at least 3 donors.

Fig. 4a demonstrates that the broad range FMO inhibitor methimazole (Phillips and Shephard, 2017) had no effect on M8OI disappearance or HO8IM and COOH7IM appearance, suggesting that FMOs are not involved in M8OI metabolism. Note, that the majority of HO8IM is further metabolised to COOH7IM and therefore the sum of both metabolites was also determined. The broad spectrum CYP inhibitors - metyrapone (Paine, 1990) or SKF525a (Franklin and Hathaway, 2008) had a moderate but significant effect on these parameters (Fig. 4b and c). Ketoconazole is a potent inhibitor of CYP3A isoforms (Greenblatt et al., 2011), although other isoforms are inhibited at higher concentrations (Eagling et al., 1998). Fig. 4d demonstrates that 10 μM ketoconazole potently blocked both disappearance of M8OI and appearance of HO8IM and COOH7IM in hepatocytes. Concentrations of ketoconazole as low as 500 nM similarly inhibited the disappearance of M8OI and appearance of HO8IM and COOH7IM in hepatocytes (data not included) implicating CYP3A forms as prominent in the monooxygenation of M8OI in human hepatocytes.

### 3.4 HO8IM is oxidised to COOH7IM by a 4-methylpyrazole- and disulfiram-inhibited alcohol dehydrogenase(s) and acetaldehyde dehydrogenase(s) respectively

Xenobiotic alcohols typically can be subject to conjugation with (phase II) water-soluble groups such as glucuronic acid or sulfate (by UDP glucuronyl transferases and sulfotransferase enzymes respectively) or oxidised to their corresponding carboxylic acids by both CYPs and/or dehydrogenases. Based on mass fragment predictions, there was no evidence for a production of a phase II conjugate at high levels (data not shown), rather the majority of H08IM was converted to a carboxylic acid via the acetaldehyde (detectable at low levels likely due to rapid enzymic conversion to the acid).

**Fig. 5:**
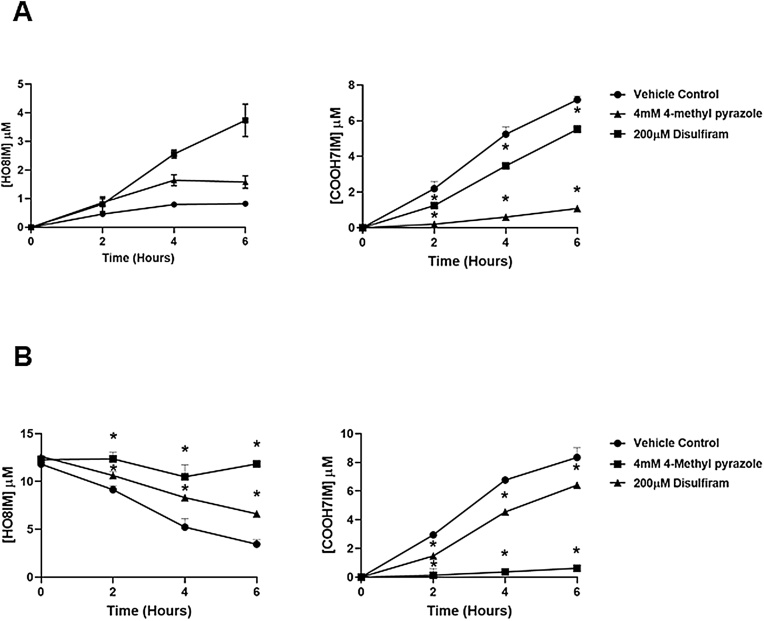
**HO8IM is primarily oxidised by an alcohol dehydrogenase(s) and acetaldehyde dehydrogenase(s) in HepaRG cells. A,** differentiated HepaRG cells were incubated with 10μM M8OI in the presence of either vehicle solvent control, 4 mM 4-methylpyrazole or 20μM disulfiram and medium levels of HO8IM (left panel) or COOH7IM (right panel) determined were examined. Data are the mean and SD of 3 separate incubations from the same batch of cells, typical of at least 3 separate experiments. **B**, differentiated HepaRG cells were incubated with 10μM HO8IM in the presence of vehicle solvent control, 4 mM 4-methylpyrazole or 20μM disulfiram and medium levels of HO8IM (left panel) or COOH7IM (right panel) determined were examined. Data are the mean and SD of 3 separate incubations from the same batch of cells, typical of at least 3 separate experiments.

Differentiated HepaRG cells were used as an additional model of human liver metabolism. Fig. 5a demonstrates that both metabolites - HO8IM and COOH7IM - were produced by HepaRG cells after addition of M8OI to cultures. Addition of either the alcohol dehydrogenase or acetaldehyde dehydrogenase inhibitors 4-methylpyrazole (Bestic et al., 2009; Hartman et al., 2014) and disulfiram (Bilska-Wilkosz et al., 2019) respectively resulted in higher medium levels of HO8IM and lower levels of COOH7IM metabolites compared to control cell cultures (Fig. 5a). Addition of HO8IM to differentiated HepaRG cells confirmed the effects of 4-methylpyrazole and disulfiram in that there was a reduction in the rate of production of the COOH7IM metabolite compared to control cell cultures (Fig. 5b). These data suggest that the metabolism of HO8IM is primarily mediated by an alcohol dehydrogenase(s) and acetaldehyde dehydrogenase(s) in HepaRG cells.

### 3.5 Metabolism of M8OI in hepatocytes from some individuals is negligible

**Fig. 6:**
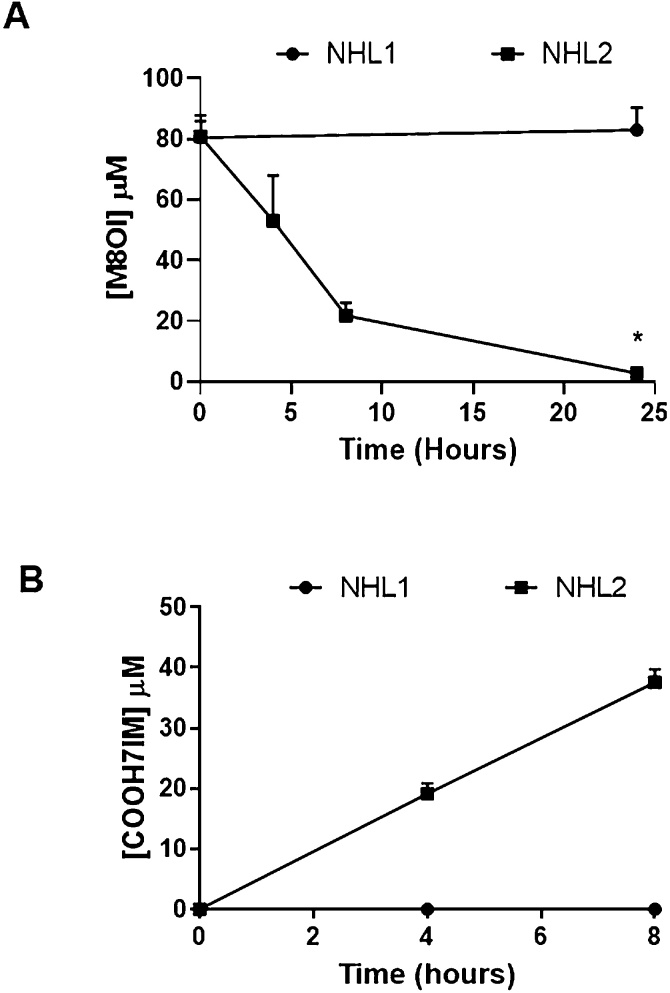
**Variability of donor human hepatocytes to metabolise M8OI.** Cultured human hepatocytes were washed and exposed to 100μM M8OI in STIM buffer as outlined in methods section. The STIM buffer concentrations of M8OI (**A**) and COOH7IM (**B**), were determined by HPLC using UV detection. Data are the mean and SD of 3 separate determinations from the same hepatocyte incubation. Data presented are those obtained from hepatocytes isolated from NHL1 and NHL2.

As part of a rapid determination of M8OI metabolism, hepatocytes from different donors were exposed to relatively high concentrations of M8OI and the disappearance from the medium and appearance of the COOH7IM metabolite examined over a 24 h period. Fig. 6 demonstrates that there may be a significant difference in the metabolism of M8OI between individuals since cells from occasional donors (e.g. NHL1) failed to significantly metabolise M8OI over a 24 h period whereas cells from the majority of donors (e.g. NHL2, typical of other preparations) cleared the medium of M8OI by 24 h, with the production of COOH7IM.

These data indicate that there are marked differences between individuals and M8OI metabolism. Since M8OI is primarily monooxygenated by a CYP3A isoform(s), it is likely that the predominant CYP3A is polymorphic.

## 4 Discussion

The results from these studies demonstrate for the first time, that there is evidence for exposure to M8OI in the general population based on a limited study in serum from 20 PBC patients and 10 controls. This study was instigated by the recent identification of M8OI in the environment (Probert et al., 2018; Leitch et al., 2020a; Oskarsson and Wright, 2019). To our knowledge, methylimidazolium ionic liquids are not used in any household product, food additive, cosmetic, pharmaceutical or pesticide (Leitch et al., 2020a). The uses of methylimidazolium ionic liquids appear to be restricted to industrial processes and applications or incorporated as undeclared components of products not expected to lead directly to human exposure (e.g. additives to batteries). However, M8OI itself is not known to be used in these processes and applications and manufacturing levels of the 5 variants of M8OI manufactured and/or imported in to the EC is low (<100 tonnes/annum). Industrial processes and applications using methylimidazolium ionic liquids appear to be restricted to smaller chain variants (ethyl and butyl analogues (Leitch et al., 2020a)), with reported levels of manufacture/importation in the EU often confidential. This study is the first, to our knowledge, to investigate the potential exposure of a methylimidazolium ionic liquid in man. Given the low use levels of M8OI, it is surprising to find detectable levels of this chemical in around 20 % of the participants in this pilot study. Accordingly, some consideration of the reliability of the measurements have been considered. In this respect, the analytical MS fragmentation data from samples was accompanied by a comparison with serum spiked with authentic M8OI (and analysed during the same run) which provides some validity to the results. Identifying a serum sample was also not reliant on the presence of a single MS fragment but on the presence of both the parent cation (M8OI^+^) and a fragment cation containing a chemical group not present in nature (MI^+^) and not observed in the majority of samples (i.e. negative samples). Furthermore, a sample was only considered to contain detectable levels of M8OI if the levels of MI^+^ were more than 50 % higher than the MS fragment levels of an endogenous serum component with similar but distinct mass to MI^+^. Accordingly, these data provide strong evidence for the presence of M8OI is selected individuals.

Based on comparison with an authentic M8OI standard (albeit, in the absence of a rigorous determination of a LOD and LOQ), an estimate for the concentration of M8OI in the serum sample with the highest concentration is 5 nM or approx1 ng/mL serum. Perfluorinated alkyl substances are a group of emerging aquatic pollutants with similar chemical properties to methylimidazolium ionic liquids with low biodegradability, high mobility in soil and water and a risk for bioaccumulation (Oskarsson and Wright, 2019). A study on serum samples collected in 1989–1990 from a US Nurses’ Health Study of 110 participants reported median perfluorooctanesulfonic acid (PFOS) and perfluorooctanoic acid (PFOA) levels of 15.86 ng/mL (range 12.27–22.00) and 4.78 ng/mL (range 3.56–6.47) respectively (Hu et al., 2019). This compares to a recent examination of serum concentrations of perfluoroalkyl acids in 200 Swedish school children in a region with low level drinking water contamination. This study determined that median serum perfluorooctanesulfonic acid (PFOS) levels were 3.3 ng/mL (range 1.1–13) and perfluorooctanoic acid (PFOA) levels 1.6 ng/mL (range 0.60–3.3) (Glynn et al., 2020). Accordingly, the serum levels of M8OI and other methylimidazolium ionic liquids are lower than these common perfluorinated alkyl substances but suggest there may be potential in the future to reach similar levels based on predicted increased use (Oskarsson and Wright, 2019).

The exposure route to M8OI in man is likely to be oral, through contamination of soil and water leading to presence in food and/or drinking water. However, there is currently no evidence to support that this may be the case. *In silico*, M8OI is predicted to be absorbed to a limited extent and to have low bioavailability in man (in contrast to smaller chain variants) (Young et al., 2020) and oral studies in mice indicate excretion of M8OI in urine and bile, metabolism to the COOH7IM metabolite and low M8OI bioavailability (Young et al., 2020). The evidence presented in this paper – based on both organ perfusion and metabolic studies with hepatocytes in vitro - suggest comparable M8OI toxicokinetics to mice in man. Should this be the case for the general population, the detection of systemically available M8OI in such a small cohort suggests either both high and widespread exposure to M8OI in the general population or to some markedly variable M8OI toxicokinetics within the human population. Given the use levels of M8OI and the absence of metabolites of M8OI in sera in those individuals positive for M8OI, the latter is more likely the case. Although the majority of donor human hepatocytes were capable of metabolising M8OI, hepatocytes from one donor showed almost no metabolism of M8OI. The reason for this limitation in M8OI metabolism has not been fully determined but may be associated with low levels of hepatocyte CYP3A in this individual (data not included). This observation could therefore account for the presence of systemic M8OI in some individuals in the absence of M8OI metabolites given the likely low levels of exposure to this chemical. A more extensive biomonitoring study would help to confirm this hypothesis. Given the high use of other methylimidazolium ionic liquids, consideration should also be given to examining exposure to these also.

The results from these studies further demonstrate for the first time that a proportion of M8OI and COOH7IM in liver perfusates is excreted in bile and that production of COOH7IM is dependent on cytochromes P450 monooxygenation followed by alcohol and acetaldehyde dehydrogenase oxidation.

However, a number of additional limitations in the study should be highlighted in particular, with regard to the dispositional and metabolic and aspects. Although in some cases, transplantable livers became available for research due to the lack of a suitable recipient, they were also rejected for transplant as they were in some cases, deemed steatotic. It should be mentioned that in the former case, livers were often in cold storage for longer. Thus, overall organ viabilities and/or functionalities may have impacted on the ability of perfused livers to model the handling of M8OI disposition and metabolism of M8OI. Additionally, donors were often on a range of drugs that are known to induce xenobiotic metabolising enzymes prior to death. It is also well known that changes (primarily a rapid loss) in xenobiotic metabolising enzymes are initiated during perfusion (Padgham and Paine, 1993) and continue to fall during culture, even in intact tissue (Wright and Paine, 1992; Padgham et al., 1993). Accordingly, the ability of perfused tissue and hepatocytes in culture to fully model M8OI metabolism in man is always in question. These issues are also relevant to HepaRG cells, which show reduced xenobiotic metabolising enzyme responses in the simple culture model used in our studies (see Ware et al., 2021). Finally, it should be noted that the concentrations of M8OI employed in perfusions and hepatocyte incubations were in considerable excess to the serum concentration detected in some individuals. Therefore, the disposition and metabolism studies may have limited relevance to that which may occur at the serum-detectable levels.

M8OI (and potentially smaller chain methylimidazolium ionic liquids which show more structural similarity to lipoic acid) are just one of a number of proposed potential triggers for PBC. Much of the evidence for any trigger as causative for PBC is limited to associations and in the absence of a clear cause and effect in an animal model, it remains challenging to fully dissect the mechanism(s) that lead to PBC. In this respect, it should not be concluded that M8OI is a significant risk for PBC causation. However, this study does suggest that the human population may already be exposed to methylimidazolium ionic liquids. Furthermore, it cannot be excluded – based on metabolism and incorporation of COOH7IM in place of lipoic acid in PDC-E2 – that M8OI and other related methylimidazolium ionic liquids have the potential to trigger PBC in exposed and susceptible individuals.

This paper therefore supports the hypothesis that there remains a hazard in man for M8OI and other related methylimidazolium ionic liquids in that they absorbed by hepatocytes and metabolised to COOH7IM. Both M8OI and COOH7IM are excreted in the bile and therefore delivered to the portal tract progenitor and/or cholangiocytes. These cells are therefore exposed to both the inducer of apoptosis and a chemical species capable of giving rise to a loss of tolerance to PDC-E2 respectively.

## Funding

This work was funded by a grant from the LIVErNORTH charity (to A.C.L and M.C.W.), the 10.13039/501100011856MRC (in the form of an ITTP studentship supporting A.C.L.) and supported by the Newton-Mosharafa Fund (in the form of a studentship supporting T.M.A); the 10.13039/501100000272National Institute for Health Research via the Health Protection Research Unit (NIHR HPRU) in Chemical and Radiation Threats and Hazards in partnership with Public Health England (PHE) and the Blood and Transplant Research Unit (NIHR BTRU) in Organ Donation and Transplantation. The views expressed are those of the authors and not necessarily those of the NHS, the NIHR, the Department of Health or Public Health England. The assistance of Sam Tingle and Lucy Bates is acknowledged.

## Declaration of Competing Interest

The authors declare that they have no known competing financial interests or personal relationships that could have appeared to influence the work reported in this paper.
