# Supplementary figures and images for: The methylimidazolium ionic liquid M8OI is detectable in human sera and is subject to biliary excretion in perfused human liver
Source: Toxicology. 2021 Jul;459:None. doi: 10.1016/j.tox.2021.152854 (PMC8366605; doi:10.1016/j.tox.2021.152854)

Supplementary data

**Supplementary Figure 1:** Analytical data for the synthesised COOH7IM.


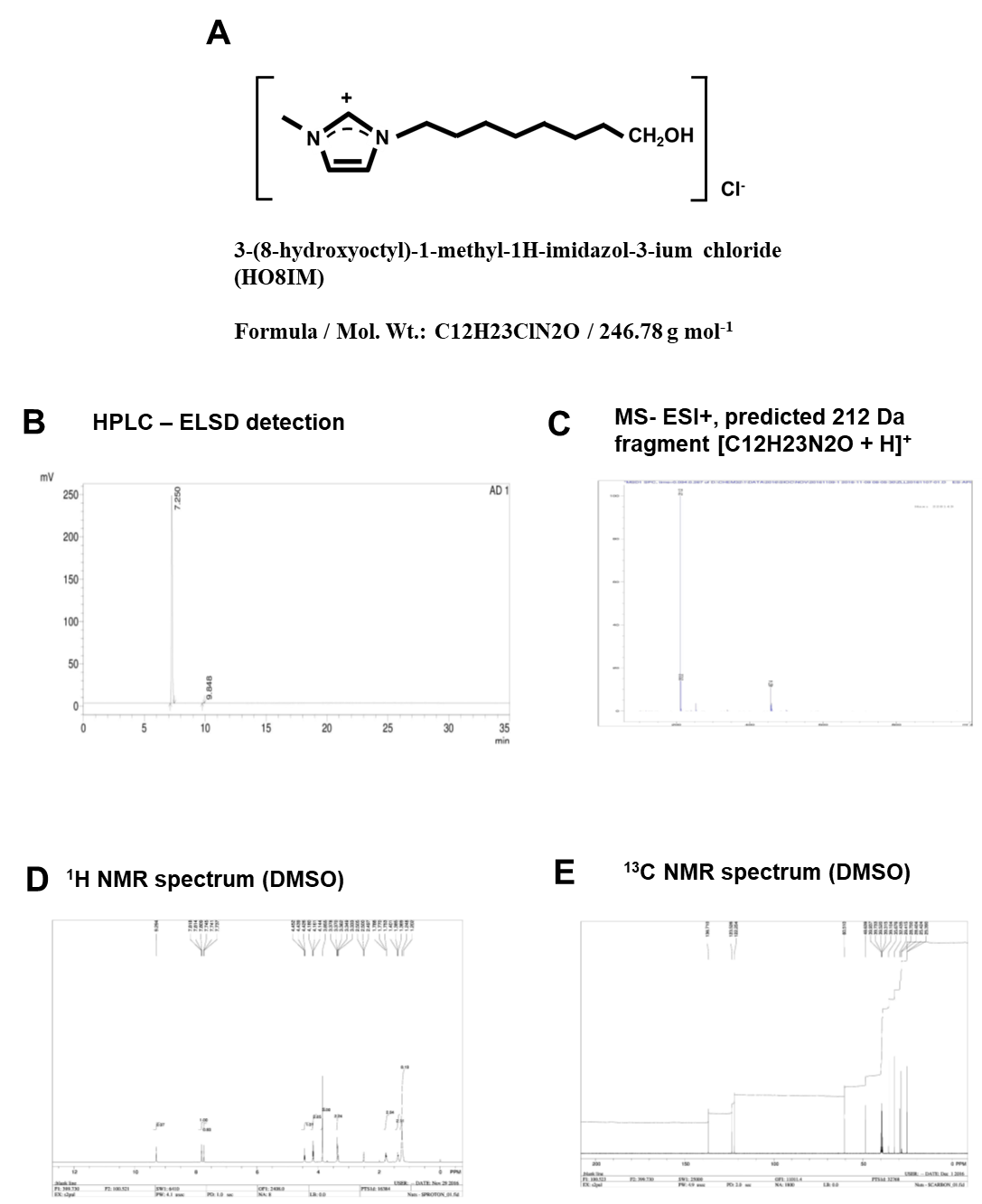

Supplement: Supplementary file 1 [file mmc1.doc]
